# Anterior Hyperfunction by Mandibular Anterior Teeth: A Narrative Review

**DOI:** 10.3390/healthcare11222967

**Published:** 2023-11-15

**Authors:** Yoichiro Ogino, Yasunori Ayukawa

**Affiliations:** 1 Section of Fixed Prosthodontics, Division of Oral Rehabilitation, Faculty of Dental Science, Kyushu University, Fukuoka 812-8582, Japan; ayukawa@dent.kyushu-u.ac.jp; 2 Section of Implant and Rehabilitative Dentistry, Division of Oral Rehabilitation, Faculty of Dental Science, Kyushu University, Fukuoka 812-8582, Japan

**Keywords:** anterior hyperfunction, combination syndrome, mandibular anterior teeth, occlusal trauma, conventional removable prostheses, implant-supported prostheses

## Abstract

“Combination syndrome”, defined by Kelly in 1972, is a challenging condition observed in a patient with an edentulous maxilla and a partially edentulous mandible with only mandibular anterior teeth. “Anterior hyperfunction syndrome” is regarded as a synonym of combination syndrome and was first described in 1994. Although these terms have been well known, the definition of “anterior hyperfunction” has not been described yet. This narrative review focused on anterior hyperfunction and discussed the etiology and the clinical managements. An electronic bibliographic search for this literature review was conducted in addition to the review of our clinical cases. The previous reports indicated that combination syndrome with all five features was rarely observed. The patients with anterior hyperfunction generally showed the loss of posterior occlusal supports and the loss of vertical dimension of occlusion. To manage anterior hyperfunction, these conditions should be improved using conventional removable prostheses and implant-supported prostheses. Anterior hyperfunction is attributed to mandibular anterior teeth and some interventions for mandibular anterior teeth are required in many cases. Additionally, it must be noted that implant-supported prostheses may lead to anterior hyperfunction. In conclusion, comprehensive approaches for the remaining teeth and the prostheses will be required to manage this complex condition.

## 1. Introduction

Masticatory function is generally influenced by the number, location and status of the remaining teeth and occlusal conditions [1,2]. Patients who have lost multiple teeth would be rehabilitated with some prostheses. However, some oral conditions may make it difficult to restore their oral function satisfactorily. Combination syndrome, defined by Kelly in 1972 [3], is characterized by the conditions observed in a patient with an edentulous maxilla and a partially edentulous mandible with only mandibular anterior teeth [3,4]. Combination syndrome has been known as a severe condition for occlusal rehabilitation. The features defined by “*The Glossary of Prosthodontic Terms: Ninth Edition (GPT-9)*” include the following features, which modified Kelly’s definitions [3,5]:

1. Loss of bone from the anterior portion of the maxillary ridge;
2. Hyperplasia of the tuberosities;
3. Papillary hyperplasia of the hard palate’s mucosa;
4. Supraeruption of the mandibular anterior teeth;
5. Loss of alveolar bone and ridge height beneath the mandibular removable partial denture bases.

In addition, Saunders et al. reported six other associated features [6]:

6. Loss of vertical dimension of occlusion (VDO);
7. Occlusal plane discrepancy;
8. Anterior spatial repositioning of the mandible;
9. Poor adaptation of the prostheses;
10. Epulis fissuratum;
11. Periodontal change.

These specific findings implied the destructive oral conditions and suggested the difficulties in the management of these patients.

Although the term “combination syndrome” was defined in 1972 as described above, “*The Glossary of Prosthodontic Terms*” which first introduced this term was the sixth edition (*GPT-6*), published in 1994 [7]. Interestingly, this edition first regarded “anterior hyperfunction syndrome” as a synonym. However, no definition of “anterior hyperfunction” was described in this edition and later editions. 

“Anterior hyperfunction” is supposed to express excessive occlusal force at the anterior site. This excessive occlusal force potentially results in a loss of bone from the anterior portion of the maxillary ridge, which is one of five changes defined by *GPT-9*. Considering the conditions of combination syndrome, excessive occlusal force from the remaining mandibular anterior teeth regardless of the existence of maxillary anterior teeth can be supposed. These conditions are observed in patients who have lost posterior occlusal supports and have remained mandibular anterior teeth [8]. These conditions are often observed in the clinic, compared to combination syndrome patients who have all five features defined by Kelly, which is considered to be lower ratios [9,10,11,12,13]. However, reports which present anterior hyperfunction are still lacking.

This review focused on anterior hyperfunction and the cause of combination syndrome and aimed to discuss their features and clinical managements.

## 2. Materials and Methods

A literature review was conducted to extract information about “anterior hyperfunction” and “combination syndrome”. An electronic bibliographic search using the National Center for Biotechnology Information (NCBI) Databases (PubMed), Web of Science, and MEDLINE (Ovid) was performed on 19 October 2023. The terms “anterior hyperfunction” and “combination syndrome” were used as search terms. Additional handsearch of some articles was made from older references and using some features of “anterior hyperfunction” and “combination syndrome”. All articles must have been published in English. In addition to the literature review, a review of our clinical cases was also presented to discuss the features of “anterior hyperfunction”.

## 3. Results

### 3.1. An Electronic Bibliographic Search and Article Selection

An electronic bibliographic search using PubMed, Web of Science, and Ovid identified 44, 79, and 44 articles, respectively. After removing duplicate articles, articles not written in English, and conference abstracts, the titles and abstracts of 74 articles were reviewed and 49 articles were related to “anterior hyperfunction” and/or “combination syndrome”. Most articles were published about “combination syndrome”, not “anterior hyperfunction”, and we decided to describe it as a narrative review. These included three literature reviews [14,15,16], one systematic review [17], and one systematic review and meta-analysis [18]. In addition, three articles were added by handsearch. However, only one article included the key word “anterior hyperfunction” in the title [19] and another article included “anterior hyperfunction syndrome” [20]. Thus, this narrative review was conducted to discuss anterior hyperfunction using the previous reports about combination syndrome and a review of our clinical cases.

### 3.2. Epidemiology and Etiology

As described above, combination syndrome is defined in patients who have all five features, as proposed by Kelly [3]. These features are usually attributed to an edentulous maxilla and a partially edentulous mandible with only anterior teeth, meaning no occlusal supports with remaining natural teeth. However, patients with all five features are observed rarely (<10%), according to the previous reports [9,10,11,12,13]. Anterior hyperfunction is caused by the loss of posterior occlusal supports and the remaining anterior mandibular teeth mainly. Consequently, findings or symptoms proposed by Kelly [3] and Saunders et al. [6], which are #1–#11 as described above, can be observed in anterior hyperfunction patients in whole or in part. This means that anterior hyperfunction causes one or more symptoms. These symptoms generally make the intervention difficult. Maxillary anterior bone loss leads to poor support in maxillary removable prostheses and makes implant placement complicated or impossible. Papillary hyperplasia also leads to the displacement of maxillary removable prostheses. Supraeruption of mandibular anterior teeth and loss of VDO result in insufficient space for the prostheses. Fabricated prostheses do not have enough thickness or strength to support occlusal force from mandibular anterior teeth. Occlusal plane discrepancy and anterior spatial repositioning of the mandible may induce biomechanical problems and result in unbalanced occlusion. All of these are the causes of poor adaptation of the prostheses. 

In 2007, Tolstunov proposed the classification of combination syndrome [8] and suggested that the patients who lost occlusal supports (Eichner C) and had mandibular anterior teeth might present the features of combination syndrome. In other words, these occlusal statuses demonstrate anterior hyperfunction, resulting in the occurrence of some features of combination syndrome. The occlusal statuses proposed by Tolstunov are as follows [8]:

1. Class I
  Maxilla: completely edentulous alveolar ridge;
  Mandible:
  Modification 1 (M1): partially edentulous ridge with preserved anterior teeth only;
  Modification 2 (M2): stable ‘‘fixed’’ full dentition (natural teeth or implant-supported crowns/bridges);
  Modification 3 (M3): partially edentulous ridge with preserved teeth in anterior and one posterior region.
2. Class II
  Maxilla: partially edentulous alveolar ridge with teeth present in both posterior regions, edentulous and atrophic anterior region;
  Mandible:
  Modifications are the same as in Class I (M1, M2, and M3).
3. Class III
  Maxilla: partially edentulous alveolar ridge with teeth present in one posterior region only and edentulous and atrophic anterior region
  Mandible: modifications are consistent with Class I and II (M1, M2, and M3).

**Figure 1:**
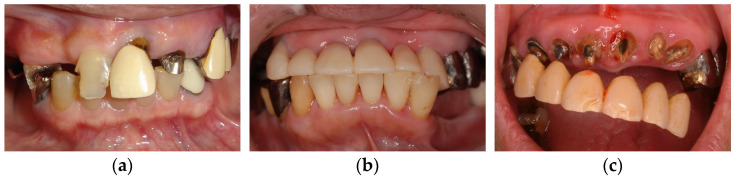
The effects of anterior hyperfunction on maxillary anterior teeth: (**a**) decay of tooth crowns; (**b**) mobile or flared maxillary anterior teeth; (**c**) troubles of prostheses in maxillary anterior teeth. Source: the authors.

In clinical cases, anterior hyperfunction is also occasionally observed in patients classified as Eichner B4 (only anterior occlusal contacts), meaning the patients with anterior teeth in both jaws and without any posterior occlusal supports. In Eichner B4 patients, the effects of anterior hyperfunction on maxillary anterior teeth are as follows (Figure 1):

1. Decay of tooth crowns;
2. Mobile or flared maxillary anterior teeth;
3. Troubles of prostheses in maxillary anterior teeth.

Decay of tooth crowns is attributed to anterior hyperfunction or excessive occlusal force. In addition, a loss of posterior supports and loss of VDO also may be associated. However, in our clinical cases, these teeth tended to have comparatively healthy periodontium. On the contrary, mobile or flared maxillary anterior teeth are attributed to impaired periodontium in addition to occlusal force or anterior hyperfunction. Troubles with prostheses in maxillary anterior teeth including tipping of facing materials and crown detachment occur due to excessive occlusal force and sequential cement wash-out. In addition, excessive occlusal force may result in tooth fracture. Secondary caries following cement wash-out and/or abutment tooth fracture may be associated with crown detachment. 

The symptoms of combination syndrome at the edentulous maxillary anterior site may be associated with the extraction of damaged maxillary anterior teeth in Eichner B4 patients with anterior hyperfunction and subsequent uncontrollable anterior hyperfunction. Especially in Eichner B4 patients, excessive occlusal force at the anterior site is known as occlusal trauma, which is exactly anterior hyperfunction in our opinion. Occlusal trauma is closely associated with the migration of maxillary anterior teeth (flared teeth) and bone resorption. Regarding occlusal trauma, two types have been well known; primary occlusal trauma and secondary occlusal trauma [21,22]. Primary occlusal trauma is defined as damage from excessive occlusal force applied to healthy periodontium. In contrast, secondary occlusal trauma is defined as damage from normal occlusal force applied to diseased or impaired periodontium. Particularly, Eichner B4 patients with impaired maxillary anterior teeth often exhibit excessive occlusal force from mandibular anterior teeth and the damage is more severe than in the patients with stable posterior occlusal supports. This results in the disruption of periodontium, migration of maxillary anterior teeth, and bone resorption. Additionally, these patients also present a loss of VDO. This also can cause anterior hyperfunction. This condition often makes the intervention difficult and induces a more complex occlusal condition. When the patients lose their posterior occlusal contacts, it is important to observe maxillary anterior teeth to find anterior hyperfunction and to prevent further occlusal disruption which leads to combination syndrome [22].

### 3.3. Clinical Management

**Figure 2:**
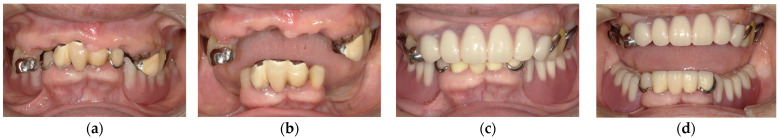
Occlusal plane discrepancy due to mandibular anterior teeth: (**a**) pre-treatment: occlusal plane discrepancy attributed to mandibular anterior teeth (intercuspal position); (**b**) pre-treatment: state of mouth opening; (**c**) post-treatment: adjustment of occlusal plane (intercuspal position); (**d**) post-treatment: state of mouth opening. Source: the authors.

Whereas anterior hyperfunction is induced by multiple factors, a loss of posterior occlusal supports and a loss of VDO are considered as primary factors. To prevent and manage anterior hyperfunction, posterior occlusal supports and VDO should be confirmed and must be reestablished if they are disrupted. In addition, supraeruption of the mandibular anterior teeth or inappropriate length of mandibular anterior teeth and subsequent occlusal plane discrepancy may be observed. It is strongly recommended to improve them to manage anterior hyperfunction effectively (Figure 2).

#### 3.3.1. Prevention of Anterior Hyperfunction and Combination Syndrome 

Patients who have lost posterior occlusal supports and have mandibular anterior teeth, such as Eichner B4, C1, or C2 patients, may develop anterior hyperfunction. Furthermore, combination syndrome shows five severe features and is an extremely difficult condition to treat. It is important to prevent the development of these features [3]. The key point must be occlusal contact at the anterior site and dentists or prosthodontists should assess occlusal force from the mandibular anterior teeth. As described above, anterior hyperfunction can be regarded as occlusal trauma, and it is critical to avoid changing anterior occlusal force into primary or secondary occlusal trauma. After reconstruction of posterior occlusal supports using conventional removable prostheses or implant-supported prostheses, some interventions for anterior teeth such as splinting and occlusal adjustment may be considered [6]. Conventional removable prostheses should be fabricated and delivered properly after the establishment of VDO. It also includes the coverage of the basal seat beneath distal extension bases in mandibular removable prostheses [6]. These may play an important role in maintaining occlusal supports and VDO. After the delivery of prostheses, the previous report recommends a meticulous follow-up care and maintenance protocol due to variable conditions [16]. 

#### 3.3.2. Clinical Management of Anterior Hyperfunction and Combination Syndrome Using Conventional Removable Prostheses

When anterior hyperfunction is already observed in patients, the reason should be considered carefully. Similarly to the prevention of anterior hyperfunction, considerable attention should be paid to posterior occlusal supports and VDO. When anterior hyperfunction is already confirmed, drastic interventions are required to provide posterior occlusal supports and to minimize occlusal force in maxillary anterior teeth [22]. The patients’ remaining maxillary anterior teeth, anterior hyperfunction or occlusal trauma by mandibular anterior teeth may influence maxillary anterior teeth negatively, but rarely in remaining mandibular anterior teeth. If no teeth are found in maxillary anterior sites, anterior occlusal contacts should be carefully examined through vertical displacement or rotation of maxillary removable prostheses and bone resorption at the anterior portion of the maxillary ridge and subsequent papillary hyperplasia. 

**Figure 3:**
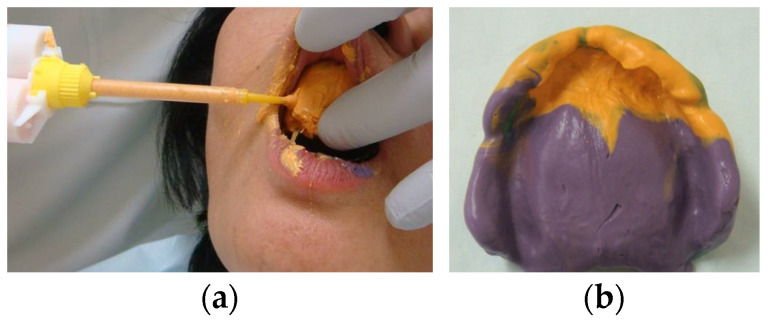
Impression procedure for flabby hyperplastic tissues: (**a**) injecting a high-flow impression material for the impression of flabby hyperplastic tissues; (**b**) final maxillary impression. Source: the authors.

**Figure 4:**
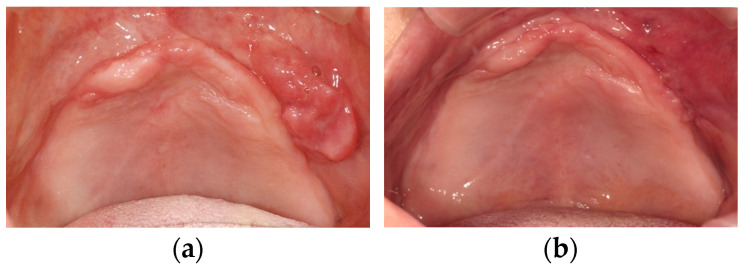
Surgical management of denture fibroma: (**a**) pre-resection; (**b**) post-resection. Source: the authors.

Management of anterior hyperfunction using conventional removable prostheses depends on the achievement of stability of the denture base and stable occlusion, especially in posterior sites, to eliminate anterior occlusal force. In the intervention for maxilla, although maxillary anterior teeth may resist anterior hyperfunction after some interventions such as splinting or occlusal adjustment and the delivery of a maxillary removable prosthesis, consecutive careful observation is required. If severe secondary occlusal trauma is observed in maxillary anterior teeth with impaired periodontium, complete dentures following extraction of these teeth or overdenture on the roots of these teeth may be considered. In patients who have already lost maxillary anterior teeth, flabby hyperplastic tissues, which is one of five features proposed by Kelly in combination syndrome patients, may be present [3]. Flabby tissues beneath the removable denture base are compressed and deformed and may cause instability of a removable denture. A special tray cut away from flabby tissues can be used for the impression of maxilla to eliminate the mobility of these tissues during the impression [23,24,25] and the high-flow impression material is used effectively to avoid impression pressure for the maxillary anterior site (Figure 3). When severe flabby tissues or denture fibroma are present in the maxillary anterior region, surgical intervention (resection of these tissues) can be a treatment option to enhance the stability of maxillary complete dentures (Figure 4) [20]. 

In the mandible, a well-functioned bilateral distal extension removable partial denture can contribute to the management of anterior hyperfunction. This is the most important management. The design should be rigid and should cover the basal seat beneath the distal extension bases properly. Prior to the fabrication of removable partial dentures, a horizontal plane of occlusion and VDO must be decided. This procedure may include intervention for the mandibular anterior teeth. Jameson suggested the “linear occlusion concept”, with the establishment of a horizontal plane of occlusion to minimize anterior occlusal contact [26,27]. Three distinct treatment procedures for mandibular anterior teeth to manage anterior hyperfunction and combination syndrome were also proposed, either individually or in combination [27]:

1. Establishment of VDO with new restorations;
2. Alteration of existing restorations to the desired VDO;
3. Modification of existing natural teeth without restorations.

**Figure 5:**
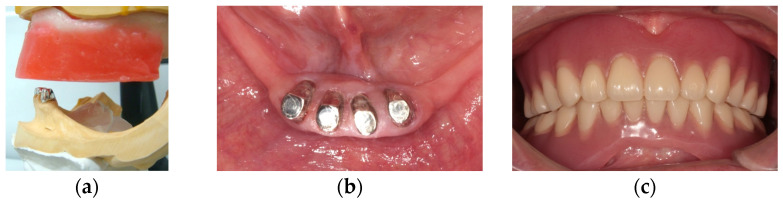
A mandibular overdenture with attachments (magnet attachments) supported by mandibular anterior teeth: (**a**) keepers of magnet attachments in mandibular anterior teeth; (**b**) post-cementation of keepers of magnet attachments in mandibular anterior teeth; (**c**) a maxillary complete denture and a mandibular overdenture at intercuspal position. Source: the authors.

These procedures are supposed to be effective to form linear occlusion and a proper horizontal plane of occlusion or proper length of mandibular anterior teeth. A crown shortening procedure is meaningful to control anterior hyperfunction and to create denture space. In the posterior sites, remarkable bone loss may be observed, and this makes definitive impression for a removable partial denture difficult. To acquire well-fitness of a denture base, the altered cast technique can be an optimal method [23]. Mandibular overdenture following shortening mandibular anterior teeth length can be available to manage anterior hyperfunction or excessive occlusal force. In mandibular overdenture cases, the use of attachments can be recommended to enhance the stability and retention (Figure 5).

**Figure 6:**
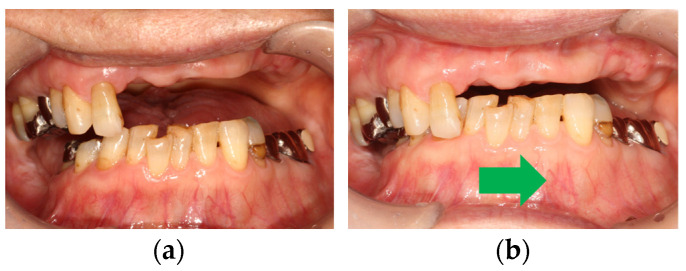
Lateral shift of mandible after first teeth contacts: (**a**) first teeth contact; (**b**) mandibular lateral shift (left side) of mandible after first contact (green arrow). Source: the authors.

Maxillomandibular registration is an important procedure to manage anterior hyperfunction and combination syndrome. To control anterior hyperfunction, the absence of anterior tooth contacts is key for the stability of maxillary removable prosthesis [27]. When patients have flabby hyperplastic tissues, the mobility of the record base with an occlusion rim should be examined carefully, even if a specific procedure was conducted during the maxillary impression described above. Flabby tissues at the anterior site enhance vertical displacement or rotation, and it is better to avoid or minimize occlusal force at the anterior site during maxillomandibular registration [23]. Daher et al. introduced methods to obtain maxillomandibular registration and definitive impressions in a single visit [24]. This report used a maxillary impression tray with an anterior window for impression and maxillomandibular registration. This anterior window can avoid occlusal force at the anterior site because there is no occlusion rim at the anterior site. However, an anterior occlusion rim is necessary to decide the position of the maxillary anterior teeth and lip support. In anterior hyperfunction patients, anterior occlusal contact during maxillomandibular registration should be considered to avoid the displacement of the maxillary anterior occlusion rim by the mandibular anterior teeth. It is recommended that maxillomandibular registration is conducted using only posterior occlusal contacts as much as possible. Maxillomandibular registration in Eichner B4 patients should be conducted with careful attention at anterior occlusal contact. Patients with anterior hyperfunction sometimes show anterior migration of maxillary anterior teeth and reestablishment of proper VDO is essential. Anterior occlusal contact, especially at the canine, occasionally causes premature contact, inducing an improper lateral shift of the mandible; so, proper maxillomandibular registration should be considered (Figure 6).

After the delivery of conventional removable prostheses, the oral condition of patients with anterior hyperfunction tends to change easily, especially at the maxillary anterior site and mandibular posterior sites. This may result in ill-fitness of the removable denture, bone resorption, and reduction in VDO, which are the causes of anterior hyperfunction. A careful, meticulous follow-up care plan would be recommended.

#### 3.3.3. Implant Treatments for Anterior Hyperfunction and Combination Syndrome 

Implant-supported prostheses can play a critical role in the rehabilitation of patients who have lost their teeth. In patients with anterior hyperfunction or combination syndrome, it is reasonable to understand that implant-supported prostheses can contribute to their rehabilitation. In these patients, implant-supported prostheses can work effectively from two viewpoints [19]:

1. Provision of stable posterior occlusal supports by mandibular implant-supported prostheses;
2. Provision of rigid prostheses in maxillary anterior region against anterior hyper function by maxillary implant-supported prostheses.

**Figure 7:**
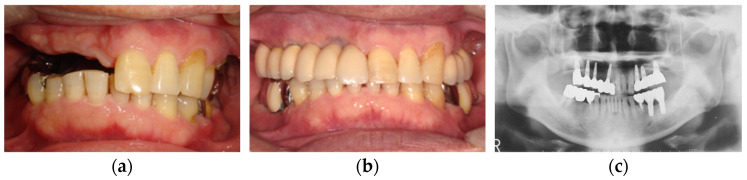
Implant-supported prostheses for the patients with anterior hyperfunction: (**a**) pre-treatment; (**b**) post-treatment; (**c**) a panoramic X-ray image of post-treatment. Source: the authors.

Although implant placement may be impossible due to the limitation of residual bone height and width in some cases, if delivered properly, implant-supported prostheses can lead to satisfactory results [8,15,17,19,28,29,30,31,32,33,34,35,36]. The reestablishment of posterior occlusal supports by implant-supported prostheses may contribute to the recovery of impaired maxillary anterior teeth by anterior hyperfunction. In addition, maxillary implant-supported prostheses opposed by the remaining mandibular anterior teeth reduce maxillary anterior and posterior alveolar bone loss compared with conventional dentures [37]. We have also experienced cases where implant-supported prostheses for patients with anterior hyperfunction contributed to the recovery of damaged maxillary anterior teeth (Figure 7). 

**Figure 8:**
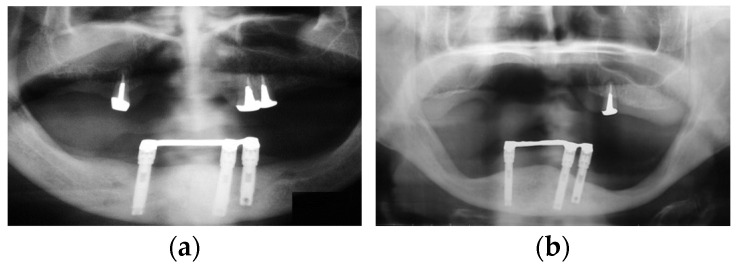
Bone resorption in a patient with implant-supported overdenture: (**a**) a panoramic X-ray image at the delivery of overdenture; (**b**) a panoramic X-ray image at 19-year follow-up. Source: the authors.

**Figure 9:**
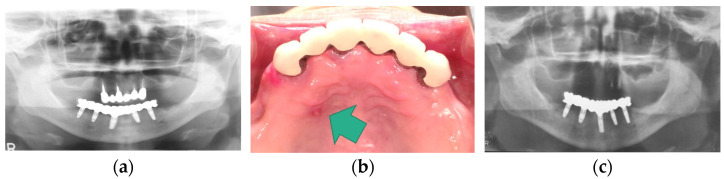
Anterior hyperfunction by an implant-supported fixed prosthesis: (**a**) a panoramic X-ray image at the delivery of prosthesis; (**b**) fistula (green arrow) attributed to root fracture; (**c**) a panoramic X-ray image after the loss of maxillary anterior teeth due to anterior hyperfunction by mandibular implant-supported prosthesis. Source: the authors.

However, there have been multiple reports that have presented mandibular implant-supported overdenture-induced anterior hyperfunction, subsequent bone resorption, and the formation of flabby hyperplastic tissues at the anterior site beneath maxillary complete denture base-opposing implants [38,39,40,41,42,43]. These reports suggested the risk of mandibular implant-supported overdentures for destructive changes on the maxillary anterior site. In fact, we experienced the negative effects of mandibular implant-supported overdentures on maxillary anterior sites and mandibular posterior sites (Figure 8). In addition, we also experienced a clinical case where a mandibular implant-supported fixed prosthesis in a patient with only maxillary anterior teeth worked as anterior hyperfunction and root fracture of the maxillary anterior tooth was confirmed. This patient’s case resulted in edentulous maxilla due to sequential root fracture and the patient was rehabilitated with maxillary complete denture, unfortunately (Figure 9). These findings suggest the risk of mandibular implant-supported prostheses working as anterior hyperfunction. Meanwhile, contrary to these reports, a recent systematic review and meta-analysis revealed that no significant difference was detected in maxillary anterior bone resorption between patients with mandibular two-implant-supported overdentures and with conventional mandibular complete dentures [18]. The retention mechanism (attachment types of overdentures), the relining of the maxillary complete denture, occlusal condition, and stages of edentulism might be associated with bone resorption, and the necessity of a well-designed randomized clinical study was suggested. However, it is important to note that mandibular implant-supported overdentures or mandibular implant-supported fixed prostheses as alternatives for conventional removable prostheses may make the problems complicated depending on the interventions.

## 4. Discussion

A literature review clearly showed that combination syndrome and its related symptoms were proposed by Kelly [3] and Saunders et al. [6], and they suggested that these specific changes represented the complexities of this oral condition. They defined these symptoms in the patient with edentulous maxilla and partially edentulous mandible with preserved anterior teeth only. Following their proposals, Tolstunov presented a new classification of combination syndrome, which is composed of three classes [8]. These classes were also defined in patients without maxillary anterior teeth. The previous reports presented that combination syndrome was induced by a loss of posterior occlusal supports and a loss of VDO, mainly [6]. However, these phenomena are also observed in patients with maxillary and mandibular anterior teeth, and with excessive occlusal force from the mandibular anterior teeth. This is generally known as occlusal trauma, which is exactly anterior hyperfunction. The term “anterior hyperfunction” was first mentioned in *GPT-6* as “anterior hyperfunction syndrome”, to the best of our knowledge. The previous reports showed that the prevalence of combination syndrome (five features) was comparatively lower [9,10,11], especially in recent studies [12,13]. This may be attributed to the prevention of combination syndrome through the previous reports. However, anterior hyperfunction is often observed in patients with a loss of anterior and posterior occlusal supports and with mandibular anterior teeth, and in patients with only anterior teeth contacts (Eichner B4 patients). It is important to control occlusal force from mandibular anterior teeth with the rehabilitation of posterior occlusal supports and VDO. These are key points for the management of anterior hyperfunction.

This review indicates multiple findings or symptoms of anterior hyperfunction, as described above. A loss of posterior supports is exactly the cause of anterior hyperfunction. A loss of VDO is not only the cause of anterior hyperfunction but is also the result following migration or flaring of maxillary anterior teeth in Eichner B4 patients and gradual poor adaptation of removable prostheses due to destructive changes by anterior hyperfunction in Eichner C patients. A poor adaptation of maxillary and mandibular removable prostheses can also be the cause of anterior hyperfunction. This review suggests that considerable observation and appropriate treatment planning would be necessary to find the causes of these findings or symptoms. 

Conventional removable prostheses can manage anterior hyperfunction and combination syndrome, including some special procedures (e.g., impression for maxillary anterior site and mandibular posterior sites, interventions for mandibular anterior teeth) [23,24,25,26,27]. However, in some cases, these prostheses are not enough to maintain posterior occlusal supports longitudinally due to artificial teeth wear and bone resorption beneath distal extension bases, followed by a loss of VDO. These may result in the gradual development of anterior hyperfunction. To prevent the development of these conditions, dentists and prosthodontists need to understand these features. To control or eliminate anterior hyperfunction, a mandibular overdenture supported by mandibular anterior teeth can be a therapeutic option. This is a direct method to weaken anterior hyperfunction. To manage anterior hyperfunction with conventional removable protheses, these viewpoints are considered. 

Implant-supported prostheses can be a suitable therapeutic option to manage anterior hyperfunction and combination syndrome. Especially, implant-supported fixed prostheses for mandibular posterior sites can play a critical role in the reestablishment of posterior supports. However, teeth wear induced by the differences of occlusal surface materials should be noted. Whereas implant-supported prostheses for maxillary anterior sites are also an effective therapeutic option to support occlusal force by anterior hyperfunction, careful observations are necessary to maintain the peri-implant bone level and the prostheses (e.g., screw loosening, chipping of materials). Although implant-supported prostheses can contribute to the management of anterior hyperfunction and combination syndrome, these can be the causes of anterior hyperfunction [38,39,40,41,42,43]. Although bone resorption in maxillary anterior sites opposing mandibular two-implant-supported overdentures demonstrated no statistical differences compared to mandibular conventional complete dentures according to a recent systematic review and meta-analysis [18], this report also concluded that further studies are needed to investigate the effects of implant-supported overdentures on bone resorption and anterior hyperfunction. In our clinical opinion, the conditions and the follow-up of maxillary prostheses are also important to exert the effect of mandibular implant-supported prostheses. These include the bone condition in the edentulous maxilla, materials of artificial teeth and denture base, and occlusal condition. We’d like to propose further studies to evaluate the effect of implant-supported fixed prostheses and overdentures on anterior hyperfunction.

This narrative review presented the clinical features of anterior hyperfunction and the necessities of comprehensive clinical managements to treat this complicated condition. However, as the previous studies described, no randomized controlled trial or prospective studies to assess the effect of intervention for patients with anterior hyperfunction and combination syndrome on temporal changes and longitudinal results have been reported. Especially, the therapeutic effects of conventional removable prostheses and implant-supported prostheses are of particular interest. Future studies to propose clinical practice guidelines would be desirable.

## 5. Conclusions

Anterior hyperfunction is generally observed in patients who have lost posterior occlusal supports and proper VDO. This is a precondition and a cause of combination syndrome and is frequently observed. Although conventional removable prostheses and implant-supported prostheses can manage these complex conditions, there are some key points to rehabilitate these patients. In addition, implant-supported prostheses for edentulous mandible may contribute to developing anterior hyperfunction and combination syndrome. To manage anterior hyperfunction longitudinally, comprehensive approaches for the remaining teeth and the prostheses, and a meticulous follow-up care and maintenance protocol are recommended.

## Data Availability

The data presented in this study are available on request from the corresponding author. The data are not publicly available due to clinical cases.

## References

[1] Ogino Y., Suzuki H., Ayukawa Y., Ueno Y., Jinnouchi A., Koyano K. (2021). Masticatory performance and other oral functions in community-dwelling elderly patients without posterior occlusal support by natural teeth. J. Oral Sci..

[2] Fan Y., Shu X., Leung K.C.M., Lo E.C.M. (2023). Association between masticatory performance and oral conditions in adults: A systematic review and meta-analysis. J. Dent..

[3] Kelly E. (1972). Changes caused by a mandibular removable partial denture opposing a maxillary complete denture. J. Prosthet. Dent..

[4] Almas K., Fawad J., Smith S. (2007). Oral implantology. Glossary of implant terms. J. Oral Implantol..

[5] Ferro K.J., Morgano S.M., Driscoll C.F., Freilich M.A., Albert D., Knoernschild K.L., McGarry T.J. (2017). The Glossary of Prosthodontic Terms: Ninth Edition. J. Prosthet. Dent..

[6] Saunders T.R., Gillis R.E., Desjardins R.P. (1979). The maxillary complete denture opposing the mandibular bilateral distal-extension partial denture: Treatment considerations. J. Prosthet. Dent..

[7] VanBlarcom C.W. (1994). Glossary of Prosthodontic Terms, Edition 6. J. Prosthet. Dent..

[8] Tolstunov L.J. (2007). Combination syndrome: Classification and case report. Oral Implantol..

[9] Shen K., Gongloff R.K. (1989). Prevalence of the ‘combination syndrome’ among denture patients. J. Prosthet. Dent..

[10] Salvador M.C., do Valle A.L., Ribeiro M.C., Pereira J.R. (2007). Assessment of the prevalence index on signs of combination syndrome in patients treated at Bauru School of Dentistry, University of Sao Paulo. J. Appl. Oral Sci..

[11] Kilicarslan M.A., Akaltan F., Kasko Y., Kocabas Z. (2014). Clinical evaluation of maxillary edentulous patients to determine the prevalence and oral risk factors of combination syndrome. J. Dent. Sci..

[12] De Resende C.M.B.M., Ribeiro J.A.M., Dias K.C., Carreiro A.F.P., Do Rego M.P.P., De Queiroz J.W.N., Barbosa G.A.S., Oliveira Â.G.R.C. (2014). Signs of Combination Syndrome and removable partial denture wearing. Rev. Odontol. UNESP.

[13] Bagga R., Robb N.D., Fenlon M.R. (2019). An investigation into the prevalence of combination syndrome. J. Dent..

[14] Palmqvist S., Carlsson G.E., Owall B. (2003). The combination syndrome: A literature review. J. Prosthet. Dent..

[15] Mijiritsky E. (2007). Implants in conjunction with removable partial dentures: A literature review. Implant Dent..

[16] Tolstunov L. (2011). Combination syndrome symptomatology and treatment. Compend. Contin. Educ. Dent..

[17] Shahmiri R.A., Atieh M.A. (2010). Mandibular Kennedy Class I implant-tooth-borne removable partial denture: A systematic review. J. Oral Rehabil..

[18] Oh W.S., Oh J., Jin Q. (2020). Bone loss in the anterior edentulous maxilla opposing two-implant-supported overdentures vs complete dentures: A systematic review and meta-analysis. Quintessence Int..

[19] Ogino Y., Kihara M., Yamada J., Toriya K., Koyano K. (2015). Implant Treatments for Edentulous Maxilla with Anterior Hyperfunction. J. Oral Implantol..

[20] Korunoska-Stevkovska V., Guguvcevski L., Menceva Z., Gigovski N., Mijoska A., Nikolovska J., Bajraktarova-Valjakova E. (2017). Prosthodontic Rehabilitation of Patient with Anterior Hyper Function Syndrome. Open Access Maced. J. Med. Sci..

[21] Hallmon W.W. (1999). Occlusal trauma: Effect and impact on the periodontium. Ann. Periodontol..

[22] Greenstein G., Cavallaro J., Scharf D., Tarnow D. (2008). Differential diagnosis and management of flared maxillary anterior teeth. J. Am. Dent. Assoc..

[23] Langer Y., Laufer B.Z., Cardash H.S. (1995). Modalities of treatment for the combination syndrome. J. Prosthodont..

[24] Daher T., Dermendjian S., Morgano S.M. (2008). Obtaining maxillomandibular records and definitive impressions in a single visit for a completely edentulous patient with a history of combination syndrome. J. Prosthet. Dent..

[25] Antonelli J., Guerrero M., Georgescu M., Ortiz J. (2019). Quantifying Flabby Ridge Tissue Displacement During Impression-Making for Patients with Combination Syndrome. Compend. Contin. Educ. Dent..

[26] Jameson W.S. (2001). The use of linear occlusion to treat a patient with combination syndrome: A clinical report. J. Prosthet. Dent..

[27] Jameson W.S. (2003). Various clinical situations and their influence on linear occlusion in treating combination syndrome: A discussion of treatment options. Gen. Dent..

[28] Beals R., Lefkove M.D. (1992). Tatum custom ramus frame implant: Multiple options including treatment for combination syndrome. J. Oral Implantol..

[29] Cabianca M. (2003). Combination syndrome: Treatment with dental implants. Implant Dent..

[30] Flanagan D. (2008). Screwless fixed detachable partial overdenture treatment for atrophic partial edentulism of the anterior maxilla. J. Oral Implantol..

[31] Tolstunov L. (2009). Management of biomechanical complication of implant-supported restoration of a patient with combination syndrome: A case report. J. Oral Maxillofac. Surg..

[32] Peñarrocha M., Viña J.A., Carrillo C., Peñarrocha D., Peñarrocha M. (2012). Rehabilitation of reabsorbed maxillae with implants in buttresses in patients with combination syndrome. J. Oral Maxillofac. Surg..

[33] Piermatti J. (2013). Rehabilitation of the edentulous maxilla complicated by combination syndrome with an implant overdenture: A case report. Gen. Dent..

[34] Carlino P., Pettini F., Cantore S., Ballini A., Grassi F.R., Pepe V. (2014). Surgical and prosthetic rehabilitation of combination syndrome. Case Rep. Dent..

[35] Andreasi Bassi M., Lopez M.A., Andrisani C., Ormanier Z., Gargari M. (2016). Full arch rehabilitation in severe maxillary atrophy with palatal approach implant placement: A case report. Oral Implantol..

[36] Uram-Tuculescu S. (2019). “Combination Syndrome” in an Upper/Lower Implant Overdenture Patient: A Clinical Report. Implant Dent..

[37] Alrajhi M.S., Askar O., Habib A.A., Elsyad M.A. (2020). Maxillary Bone Resorption with Conventional Dentures and Four-Implant-Supported Fixed Prosthesis Opposed by Distal-Extension Partial Dentures: A Preliminary 5-year Retrospective Study. Int. J. Oral Maxillofac. Implant..

[38] Maxson B.B., Powers M.P., Scott R.F. (1990). Prosthodontic considerations for the transmandibular implant. J. Prosthet. Dent..

[39] Lechner S.K., Mammen A. (1996). Combination syndrome in relation to osseointegrated implant-supported overdentures: A survey. Int. J. Prosthodont..

[40] Thiel C.P., Evans D.B., Burnett R.R. (1996). Combination syndrome associated with a mandibular implant-supported overdenture: A clinical report. J. Prosthet. Dent..

[41] Gupta S., Lechner S.K., Duckmanton N.A. (1999). Maxillary changes under complete dentures opposing mandibular implant-supported fixed prostheses. Int. J. Prosthodont..

[42] López-Roldán A., Abad D.S., Bertomeu I.G., Castillo E.G., Otaolaurruch E.S. (2009). Bone resorption processes in patients wearing overdentures. A 6-years retrospective study. Med. Oral Patol. Oral Cir. Bucal.

[43] Alsrouji M.S., Ahmad R., Rajali A., Mustafa N.W.N.A., Ibrahim N., Baba N.Z. (2019). Mandibular Implant-Retained Overdentures: Potential Accelerator of Bone Loss in the Anterior Maxilla?. J. Prosthodont..

